# RETRACTED: Ullah et al. Fluorescent and Phosphorescent Nitrogen-Containing Heterocycles and Crown Ethers: Biological and Pharmaceutical Applications. *Molecules* 2022, *27*, 6631

**DOI:** 10.3390/molecules31050775

**Published:** 2026-02-25

**Authors:** Faiz Ullah, Sami Ullah, Muhammad Farhan Ali Khan, Muhammad Mustaqeem, Rizwan Nasir Paracha, Muhammad Fayyaz ur Rehman, Fariha Kanwal, Syed Shams ul Hassan, Simona Bungau

**Affiliations:** 1 Department of Chemistry, Quaid I Azam University, Islamabad 45320, Pakistan; 2 Department of Zoology, Government College University, Faisalabad 38000, Pakistan; 3 Faculty of Pharmacy, Capital University of Science and Technology, Islamabad Expressway, Islamabad 44000, Pakistan; 4 Institute of Chemistry, University of Sargodha, Sargodha 40100, Pakistan; 5 Department of Chemistry, Sub Campus, University of Sargodha, Bhakkar 30000, Pakistan; 6 School of Biomedical Engineering, Shanghai Jiao Tong University, 1954 Hua Shan Road, Shanghai 200030, China; 7 Shanghai Key Laboratory for Molecular Engineering of Chiral Drugs, School of Pharmacy, Shanghai Jiao Tong University, Shanghai 200240, China; 8 Department of Natural Product Chemistry, School of Pharmacy, Shanghai Jiao Tong University, Shanghai 200240, China; 9 Department of Pharmacy, Faculty of Medicine and Pharmacy, University of Oradea, 410028 Oradea, Romania

The journal retracts the article titled “Fluorescent and Phosphorescent Nitrogen-Containing Heterocycles and Crown Ethers: Biological and Pharmaceutical Applications” [1], cited above.

Following publication, concerns were brought to the attention of the Editorial Office regarding the relevance of several references cited in the study [1]. 

In accordance with the journal standard procedures, the Editorial Office and Editorial Board conducted an investigation and determined that a substantial proportion of the citations in the original publication lacked appropriate relevance to the content of the article. Consequently, the Editorial Board concluded that the article did not provide appropriate or adequate citations to support its findings and has therefore decided to retract this publication [1], as per MDPI’s retraction policy. 

This retraction was approved by the Editor-in-Chief of the *Molecules* journal.

The authors have been informed of this retraction and have indicated their disagreement with this decision.
